# The Molecular Function of PURA and Its Implications in Neurological Diseases

**DOI:** 10.3389/fgene.2021.638217

**Published:** 2021-03-11

**Authors:** Lena Molitor, Sabrina Bacher, Sandra Burczyk, Dierk Niessing

**Affiliations:** ^1^Institute of Structural Biology, Helmholtz Zentrum München – German Research Center for Environmental Health, Neuherberg, Germany; ^2^Institute of Pharmaceutical Biotechnology, Ulm University, Ulm, Germany

**Keywords:** PURA syndrome, amyotrophic lateral sclerosis/fronto-temporal dementia, fragile X-associated tremor ataxia syndrome, Pur-alpha, PURB, PURG

## Abstract

In recent years, genome-wide analyses of patients have resulted in the identification of a number of neurodevelopmental disorders. Several of them are caused by mutations in genes that encode for RNA-binding proteins. One of these genes is *PURA*, for which in 2014 mutations have been shown to cause the neurodevelopmental disorder PURA syndrome. Besides intellectual disability (ID), patients develop a variety of symptoms, including hypotonia, metabolic abnormalities as well as epileptic seizures. This review aims to provide a comprehensive assessment of research of the last 30 years on PURA and its recently discovered involvement in neuropathological abnormalities. Being a DNA- and RNA-binding protein, PURA has been implicated in transcriptional control as well as in cytoplasmic RNA localization. Molecular interactions are described and rated according to their validation state as physiological targets. This information will be put into perspective with available structural and biophysical insights on PURA’s molecular functions. Two different *knock-out* mouse models have been reported with partially contradicting observations. They are compared and put into context with cell biological observations and patient-derived information. In addition to PURA syndrome, the PURA protein has been found in pathological, RNA-containing foci of patients with the RNA-repeat expansion diseases such as fragile X-associated tremor ataxia syndrome (FXTAS) and amyotrophic lateral sclerosis (ALS)/fronto-temporal dementia (FTD) spectrum disorder. We discuss the potential role of PURA in these neurodegenerative disorders and existing evidence that PURA might act as a neuroprotective factor. In summary, this review aims at informing researchers as well as clinicians on our current knowledge of PURA’s molecular and cellular functions as well as its implications in very different neuronal disorders.

## Introduction

RNA-binding proteins fulfill a number of important regulatory functions for cell differentiation, cellular asymmetry, and somatic cell activities. In recent years, particular attention has been paid to the functions of RNA-binding proteins in neuronal cells where their involvement in messenger RNA (mRNA) transport and translational regulation has been linked to memory and learning as well as to neuronal disorders (Tang, 2016; Holt et al., 2019; Thelen and Kye, 2019). One of them is the so-called Purine-rich single-stranded DNA (ssDNA)-binding protein alpha (PURA, also referred to as Pur-alpha), which frequently appears in high-throughput analyses of interaction networks in neuronal tissue. PURA is co-purifying with a multitude of different potential binding partners and has been linked by several of these studies to neural pathologies. Despite a significant body of novel insights from recent studies, still very little is known about its cellular and molecular functions. Hence, a critical assessment of our current knowledge is much needed.

The PURA protein was first described to bind to ssDNA in cell culture and brain extracts (Bergemann and Johnson, 1992; Bergemann et al., 1992; Haas et al., 1993, 1995). Such early work provided evidence for the role of PURA in transcriptional control. Only more recently, its function in RNA-regulated processes has been studied in greater detail, leading to the altered perception that post-transcriptional control constitutes a central function of the PURA protein.

In this review, we provide a detailed analysis of our current knowledge on this protein and its different cellular roles in the nucleus and cytoplasm. We combine this with molecular and structural insights and provide an update of PURA’s role in neuronal disorders, such as Fragile X-associated tremor ataxia syndrome (FXTAS), the amyotrophic lateral sclerosis (ALS), fronto-temporal dementia (FTD) spectrum disorder, and the PURA syndrome. Together, the available information indicates that the importance of PURA protein for understanding neuronal disorders is still considerably underestimated.

## Homologs of Pura are Found in Different Kingdoms of Life

To date, homologs of PURA have been described in several organisms, including metazoans, such as *Drosophila melanogaster* (Graebsch et al., 2009; Aumiller et al., 2012), *Danio rerio* (Penberthy et al., 2004), and *Caenorhabditis elegans* (termed PLP-1; Witze et al., 2009), plants such as *Arabidopsis thaliana* (Tremousaygue et al., 2003), and even in bacteria like *Borrelia burgdorferi* (Graebsch et al., 2010). Surprisingly, sequence alignments (Altschul et al., 1990) suggest that PURA is lacking in fungi.

**Figure 1:**
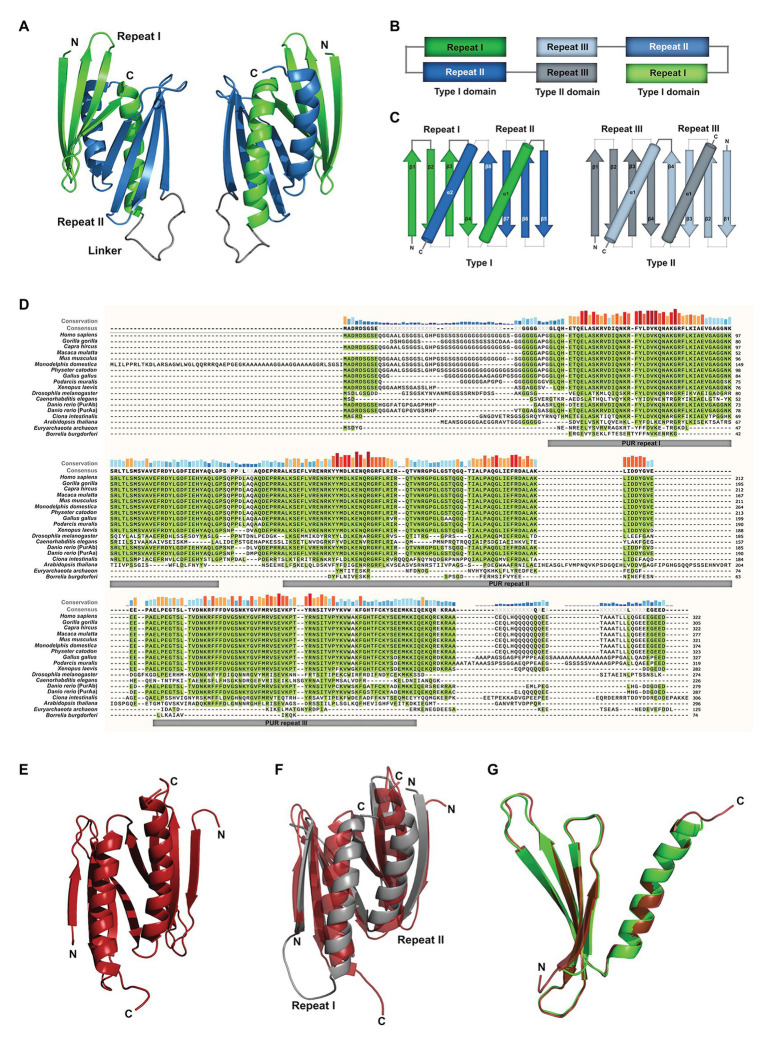
Assessment of structural and sequence conservation between PURA proteins from different species. **(A)** Cartoon backbone model of the PURA DNA-binding domain of *Drosophila melanogaster* and a view rotated 180°. **(B)** Schematic illustration of the domain organization of the PURA homodimer. **(C)** Topology diagram of the type I and type II domain of PURA. **(D)** Amino acid sequence alignment of PURA from selected species with identical amino acids highlighted in green. The sequence similarity is notably higher within the PUR repeats and decreases with evolutionary distance. The alignment was executed using the MUSCLE algorithm and program MEGAX (Hall, 2013; Mello, 2018). **(E)** Cartoon backbone model of *Borrelia burgdorferi* PURA domain forming a type II homodimer **(C)**. **(F)** Superposition of the DNA binding domain of *D. melanogaster* [shown in **(A)**] and *B. burgdorferi* [shown in **(E)**; RMSD = 2.107]. **(G)** Superposition of *D. melanogaster* PURA repeat I and *B. burgdorferi* PURA (RMSD = 0.293).

Most of the available studies have been performed with mouse PURA, which is almost identical to its human ortholog (>99% protein sequence identity; Ma et al., 1994). Early work already recognized the presence of repetitive sequence elements in the protein, which were defined as class I and class II motifs (Bergemann and Johnson, 1992). However, crystal structure determination of PUR domains from *D. melanogaster* (Figure 1A) showed an insufficient correlation of these motifs with the domain folds. Furthermore, no defined function could be assigned to them. With improved bioinformatic tools and structural information, three so-called PUR repeats were defined that show sequence conservation in all PUR proteins (Graebsch et al., 2009, 2010) and correlate well with its folded entities (Figures 1A,B). The crystal structure of PUR repeats I and II from *D. melanogaster* PURA revealed that two PUR repeats interact with each other to fold into a stable PUR domain (Graebsch et al., 2009). With a conserved β-β-β-β-*α* topology, each of the two PUR repeats folded into a four-stranded beta sheet, followed by a single alpha helix. Such a PUR domain can either be built from a single peptide chain with a β-β-β-β-α-linker- β-β-β-β-α topology (type I) or from two identical peptides with β-β-β-β-α fold, forming an inter-molecular homodimer (type II; Figures 1B,C; Janowski and Niessing, 2020). In both cases, the α-helices swap between both β-β-β-β-α repeats. In PURA, the first and second PUR repeats form a type I domain, whereas at the C-terminus a PUR domain is assembled by the third repeats of two different PURA molecules (Figures 1B,C; Graebsch et al., 2009; Weber et al., 2016).

PUR repeats and domains have also been found in bacteria (Graebsch et al., 2010). Although almost no protein sequence conservation can be found for instance between the PUR repeat of PURA from *B. burgdorferi* and metazoan (identities of 21, 23, and 22% to repeats I, II, and III of human PURA, respectively; Figure 1D; Graebsch et al., 2010), its structure is almost identical to the PUR domain from *D. melanogaster* (Figures 1E–G). Thus, PUR proteins provide a good example that the same fold can be achieved from very different sequences (Doolittle, 1994).

Albeit showing no sequence conservation to other classes of nucleic-acid binding proteins, the domain fold of PUR domains is found in a large variety of proteins, which all belong to the PC4-like family of proteins (Janowski and Niessing, 2020). They are named after the first high-resolution structure of this fold (PDB ID: 1pcf) from the human replication and transcription cofactor PC4 (Brandsen et al., 1997). Interestingly, the proteins of this family contain exclusively PC4-like domains and to some extent unstructured regions but lack any other types of domains. This observation is unusual at least for RNA-binding proteins, which very often contain combinations of different domain types to expand their molecular function (Lunde et al., 2007).

**Figure 2:**
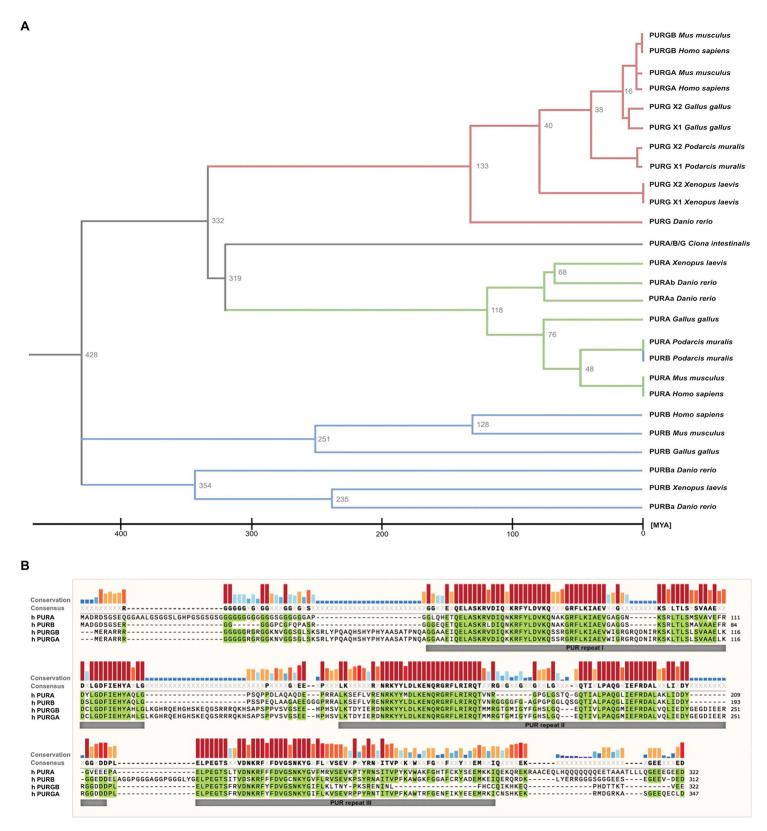
Sequence similarities between PUR-family proteins. **(A)** Phylogenetic tree of the PUR-proteins of selected species. The tree shows evolutionary time on the *X* axis and indicates a differentiation of the PUR proteins between 428 and 319 Million Years Ago (MYA). The tree was inferred using the RelTime method and was computed using one calibration constraint. Branch lengths were calculated using the Maximum Likelihood (ML) method and the JTT matrix-based substitution model. A discrete Gamma distribution was used to model evolutionary rate differences among sites. The phylogenetic analysis was performed with the program MEGAX (Hall, 2013; Mello, 2018). **(B)** Amino acid sequence alignment of human PURA, PURB, PURG variant A, and PURG variant B. Sequence identity is highlighted in green. Whereas human PURA and PURB proteins share 70% sequence conservation, PURG has 48% identity to PURA and thus is more divergent. For all paralogs, sequence similarity is notably higher within the PUR repeats. The alignment was executed using the MUSCLE algorithm and program MEGAX.

While in most organisms only one *PURA* gene exists, some species such as *D. rerio* contain two gene copies (Figure 2A). *PURA* is also unusual because it belongs to the 3% of human genes that lack introns (Grzybowska, 2012). Since alternative splicing of intron-containing genes is an important factor to modulate and expand functional properties of a given protein, this limitation indicates that there has been evolutionary pressure preventing genomic rearrangements of the *PURA* gene. This hypothesis is consistent with the observation that mutations in almost any part of the PURA protein result in the full spectrum of the human PURA syndrome (see below).

Vertebrate genomes, such as mouse, rat, and humans usually contain two additional paralogs of the *PURA* gene, termed *PURB* und *PURG*. All paralogs contain PUR-repeats as characteristic protein-sequence elements (Graebsch et al., 2009, 2010; Figure 2B).

Of note, phylogenetic analysis of the PUR protein amino acid sequences suggests that PURB emerged prior to PURA and PURG (Figure 2A) and thus is the likely founding member of the PUR family of proteins. Furthermore, the PURB orthologs share a higher conservation between species than PURA and PURG (Figure 2B).

## Molecular Functions of Pura

PURA was initially found in HeLa cells and described as an ssDNA binding factor (Bergemann and Johnson, 1992; Bergemann et al., 1992). Shortly afterwards, it was also isolated from mouse-brain lysates and shown to bind to a promoter region of the myelin basic protein (Haas et al., 1993, 1995). One of the first indications that PURA also interacts with RNA came from a study with quail embryonic fibroblasts, where RNase treatment of nuclear extracts resulted in a loss of association of PURA with larger complexes (Herault et al., 1995). Concurrently, interactions of PURA with the RNAs of the HI and JC viruses were also described (Tada and Khalili, 1992; Chen et al., 1995; Chepenik et al., 1998), indicating that this protein might act for instance as host factor for HIV propagation (White et al., 2009).

**Figure 3:**
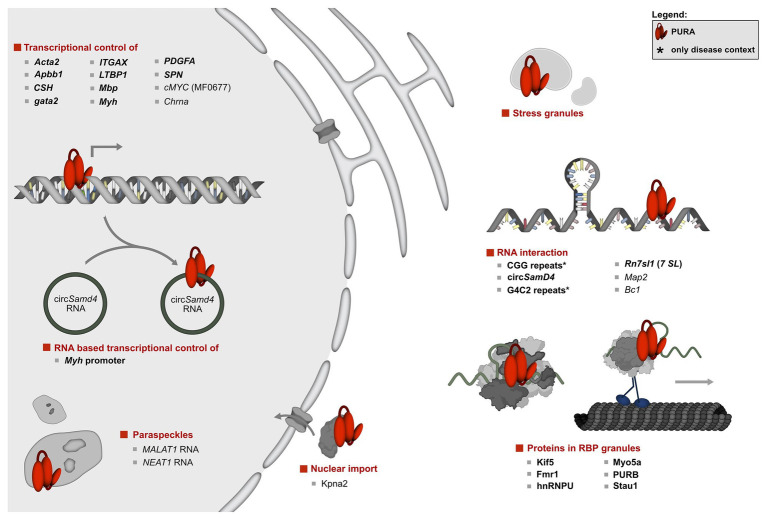
Overview of the molecular pathways of PURA and its known interactions. To date, the only protein interaction of PURA for which experimental evidence indicates a direct binding event is KIF-5 (marked in bold type). DNA and RNA targets that were confirmed by *in vitro* binding as well as by functional assays or direct correlation with observed effects in animal model organisms were considered to be validated targets (marked in bold letters). They include the human *CD43* gene promoter (Shelley et al., 2001; Da Silva et al., 2002), *FE65* promoter (Zambrano et al., 1997), *Gata2* promotor region in zebrafish (Penberthy et al., 2004), *MB1* regulatory region of the *MBP* gene (Haas et al., 1995), *Mhc* promoter (Gupta et al., 2003; Ji et al., 2007; Pandey et al., 2020), *ovine placental lactogen* promotor (Limesand et al., 2004), *TGF-ß1* (Thatikunta et al., 1997; Knapp et al., 2006), and the *VSM-alpha actin* promoter (Knapp et al., 2006). Previously described DNA and RNA interactions of PURA were considered to be only candidate targets if direct binding had been shown either by *in vitro* experiments or by high-throughput analyses, but where further functional verification is lacking. Those candidate targets are displayed in regular type and include the *A-ß-PP* (Darbinian et al., 2008), the *cMyc* upstream promoter region *MF0677* (Bergemann and Johnson, 1992; Jurk et al., 1996; Ding et al., 1997; Graebsch et al., 2009; Weber et al., 2016), *nAch* receptor (Du et al., 1997), and *TNF-alpha* (Darbinian et al., 2001b). Although for the latter, a reporter assay has been published, this was done only as duplicate and lacked statistical analyses. For simplicity reasons, interaction partners shown in this figure constitute a selection. A complete list of all reported interacting partners is shown in Figure 4.

**Figure 4:**
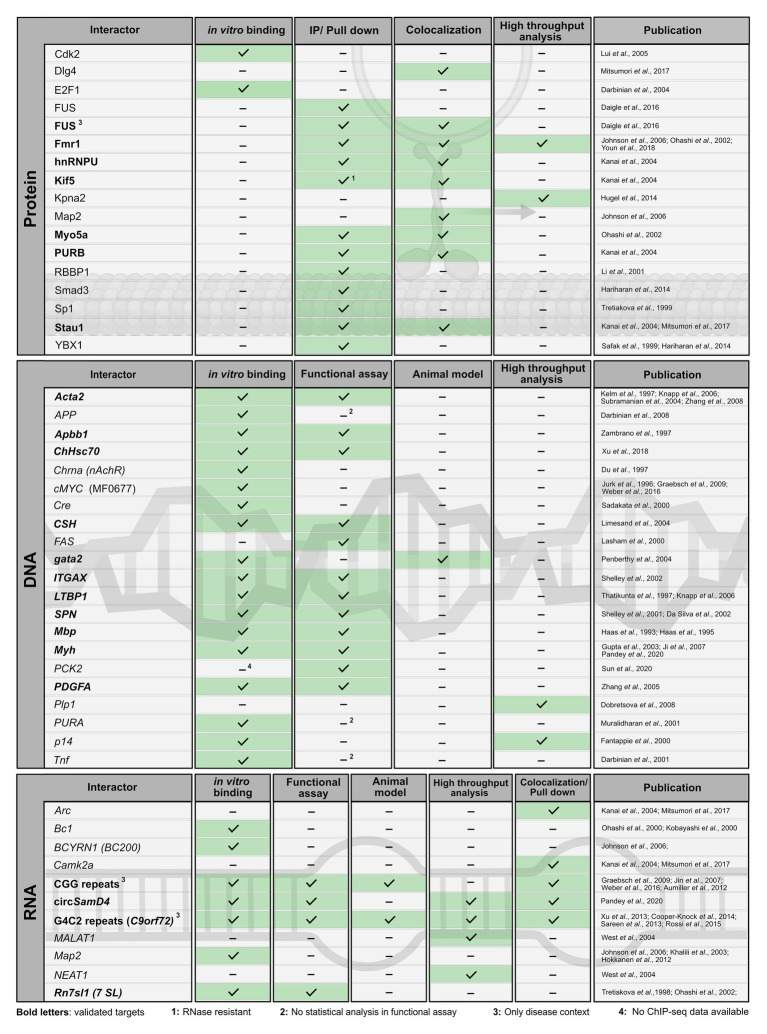
Published interaction partners of the PURA protein. For all “Interactors” (left column) the method used to observe interactions is indicated by a checkmark and green background in the respective column. Well-validated interaction partners of PURA protein are marked in bold letters. For proteins, interaction partners were defined in this study as well validated when experimental evidence is available from each of the following two categories: (i) *in vitro* binding using recombinant proteins or in immunoprecipitation/pull-down experiments from cell lysates; (ii) colocalizing with PURA in cells or identification as PURA interactor in a high throughput proteome analysis. DNA- and RNA-interaction partners are defined as well validated if *in vitro* binding by PURA as well as functional validation either by a reporter assay or in an animal model has been reported. Protein interaction labeled with “1” was shown to be RNAse resistant. Functional assays labeled with “2” lacked basic statistical analyses (i.e., SD), and hence were ignored as positive score for target validation. Interaction partners labeled with “3” have only been described in a specific disease context. For target labeled with “4” ChIP-seq data have been published (Sun et al., 2020), however without providing the original data for critical assessment.

In the past, several protein and nucleic-acid targets have been reported (Figures 3, 4). For many of them, PURA-protein fragments bearing internal deletions were created to map respective interaction sites for its binding partners (summarized in White et al., 2009; Daniel and Johnson, 2018). Unfortunately, most of these studies were done before X-ray structures of PURA were available and the respective deletions violated domain boundaries. As a consequence, such mutant versions of PURA were most likely unfolded and non-functional. For this very reason, these mapped interaction sites within PURA have to be considered with great caution for instance for proteins, such as HIV-Tat (Krachmarov et al., 1996), Polyomavirus large T-antigen (Gallia et al., 1998), Retinoblastoma protein (Johnson et al., 1995), Cdk2 (Liu et al., 2005), E2F-1 (Darbinian et al., 2004), YB1 (Safak et al., 1999), Cdk9 (Darbinian et al., 2001b), and Cyclin T1 (Darbinian et al., 2001b). Furthermore, since most reported interactions were detected by methods that do not allow to distinguish between direct and indirect interactions (Figures 3, 4) it remains to be shown whether at least some of them represent direct binding events. In particular for DNA targets of PURA, a number of cases were reported only based on observed *in vitro* interactions and thus lack a clear confirmation of their physiological relevance. Figure 4 gives an overview of the reported DNA, RNA, and protein interactions of PURA, indicating how well each target has been validated to date.

## Nuclear Targets of Pura

The first publications on PURA suggested that this protein has a strict requirement for purine-rich sequences on both, ssDNA and RNA level (Bergemann and Johnson, 1992). However, this assumption was challenged shortly afterwards (Jurk et al., 1996). Even to date, the few reported *in vitro* binding assays with short defined oligonucleotides do not hint at strict sequence specificity for purines. PURA targets include for instance the TNF-alpha promoter (Darbinian et al., 2001b), tat-responsive elements within the TGFb-1 promoter (Thatikunta et al., 1997), and human ferritin gene promoter (FE65; Zambrano et al., 1997; Figure 4). In many studies, the interaction of PURA with promoter regions were shown *in vitro*, but a quantitative assessment of binding was only made in few exceptions. Furthermore, a part of the published DNA targets of PURA were classified as such exclusively by *in vitro* binding assays. In Figures 3, 4, we defined those targets as “candidates” and distinguish them from targets that have been validated in addition for instance by reporter assays or observed changes in *knock-out* mice (Figure 3; candidate targets are shown with regular letters, validated targets with bold letters). It should be noted that the vast majority of promoter interaction studies were published more than 15 years ago and were not confirmed since then. While PURA does bind GGN motifs *in vitro* in the low micro-molar concentration range, the affinity for a short sequence within the upstream promoter region MF0677 of the cMYC gene is at least an order of magnitude better (Jurk et al., 1996; Graebsch et al., 2009; Weber et al., 2016). Since this sequence does not exclusively consist of GGN repeats, it remains to be shown in what way purines play a role in binding and if a consensus binding motif can be deduced.

One important consideration for the physiological impact of nucleic acid binding by PURA is its binding preference for either ssDNA or single-stranded (ss) RNA. Surprisingly, recombinantly expressed PURA from *D. melanogaster* bound to ssDNA and RNA oligonucleotides with identical sequences with similar equilibrium dissociation constants. Furthermore, NMR chemical shift perturbation experiments showed that the same amino acids are affected in PURA upon binding to DNA and RNA (Weber et al., 2016). It indicates that PURA binds both types of nucleic acids in the same way. A co-structure of the N-terminal PUR domain of *D. melanogaster* PURA with a short ssCGG-repeat DNA further confirmed these observations by showing that the 3' position of the ribose ring does not contribute to the binding event (Weber et al., 2016). In summary, these data indicate that PURA binds ssDNA and ssRNA equally well and in a similar way. These *in vitro* results also suggest that competition between DNA and RNA could be an important feature for PURA-dependent regulation of gene expression in cells.

Indeed, a recent and comprehensive study provided direct evidence for an RNA-based transcriptional repression of the muscle myosin-heavy chain (*Mhc*) gene by PURA and PURB. Gorospe and colleagues found that the circular RNA circSamD4 binds and sequesters both PUR proteins and thereby prevents them from repressing the transcription of the *Mhc* gene (Pandey et al., 2020). This finding is consistent with previous studies showing that PURA and PURB regulate alpha- and beta-myosin heavy chains (Gupta et al., 2003; Hariharan et al., 2014). Another study showed that PURA and PURB localized to so-called paraspeckles, which are nuclear membrane-less compartments. There, PURA was detected in the interactome of NEAT1 and MALAT1 nuclear RNAs (West et al., 2014). Taken together, independent studies demonstrated that PURA interacts with nuclear RNA, potentially allowing for direct competition between DNA and RNA binding as means for gene regulation. Future work will be necessary to establish how many more genes are regulated by the PUR protein family through such RNA-/DNA-competition mechanisms. This can be investigated for instance *via* a high-throughput identification of direct DNA and RNA targets, followed by systematic motif searches. Based on our current limited knowledge it is already clear that the name of the PUR-protein family, Purine-rich ssDNA-binding protein, is likely to not fully reflect the protein’s nucleic acid-binding features.

## Cytoplasmic Targets of Pura

Early *Drosophila* oogenesis and embryogenesis are considered as prime example for the importance of post-transcriptional gene regulation (Lasko, 2020). Indeed, also GFP-tagged PURA is transported together with many RNAs into the oocyte, suggesting a role in RNA-based gene regulation (Aumiller et al., 2012). Many other RNA-binding proteins important for early development, such as Bicoid, Bruno-1, Egalitarian, Oskar, Staufen, and Vasa have been identified already a long time ago (reviewed in reference Lasko, 2020). Due to the genetic location of PURA on the rather inaccessible fourth *Drosophila* chromosome, it has not been studied comprehensively and might have escaped potential detection in systematic genomic approaches. Since to date no phenotypic data are available in *D. melanogaster*, it is certainly possible that the biological importance of PURA is underestimated for this model organism.

However, the majority of studies were performed in mice, where PURA is detected predominantly in the cytoplasm (Figure 3; Khalili et al., 2003; Johnson et al., 2006; Hokkanen et al., 2012; Rossi et al., 2015; Daigle et al., 2016; Mitsumori et al., 2017; Swinnen et al., 2018). Apart from a diffuse cytoplasmic localization PURA is also reported to localize in cytoplasmic membrane-less compartments of vertebrates, including stress granules (Daigle et al., 2016; Markmiller et al., 2018) and neuronal transport granules (Figure 3; Li et al., 2001; Ohashi et al., 2002; Kanai et al., 2004; Mitsumori et al., 2017). Furthermore, in *C. elegans*, the PURA ortholog PLP-1 localizes to posterior germ granules, where it is important for genome maintenance (Vishnupriya et al., 2020). For stress granules, PURA has even been reported to be essential for their formation (Daigle et al., 2016). There and in neuronal RNP granules, PURA co-localizes with other neuronal RNA-binding proteins, such as PURB, Staufen, hnRNPU, and fragile X mental retardation protein (FMRP; Ross et al., 1997; Ohashi et al., 2002; Kanai et al., 2004; Johnson et al., 2006). In neuronal cells, PURA also closely associates with the cargo-transporting conventional kinesin KIF5 and myosin Myo5a, which both mediate RNA transport along neurites (Ohashi et al., 2002; Kanai et al., 2004). Upon mGluR5 activation, PURA is specifically transported in a Myo5a dependent manner into dendritic spines, where it colocalizes with the synaptic factor PSD95 (Mitsumori et al., 2017). Furthermore, other PURA-associated factors, such as FUS, FMRP, and Stau1 are reported to play key roles in the development of long-term depression (LTD; Mitsumori et al., 2017). Stau1 might be important in this context as it is associated with PURA-positive granules in dendritic shafts (Mitsumori et al., 2017). On the other hand, Stau1 is vastly diminished in PURA positive granules in dendritic spines, suggesting that it escorts PURA only before reaching the final destination in spines.

These observations are also consistent with the association of PURA and PURB with BC1 RNA in mouse brain cells (Ohashi et al., 2002; Kobayashi et al., 2011). Absence of BC1 has been implicated in altered glutamatergic transmission and maladaptive behavior (Briz et al., 2017). Furthermore, it was shown that PURA can associate with MAP2 mRNA in mouse brains and is suggested to be involved in its transport (Johnson et al., 2006). This observation is in line with results from PURA *knockout* mice, where a significant overall reduction of MAP2 protein in whole brain lysates was reported (Hokkanen et al., 2012). Apart from that, MAP2 was misdistributed to the somata of neurons and Purkinje cells. This might indicate a specific reduction of local MAP2 translation at distal neurites, potentially due to impaired transport of its mRNA (Hokkanen et al., 2012).

Since most publications showed PURA to be mainly located in the cytoplasm, it also seems likely that one of PURA’s main functions is its cytoplasmic interaction with RNA. Out of all RNA-related processes, neuronal cytoplasmic RNA transport has been associated most convincingly with a PURA function so far. Furthermore, this function fits very well with neurodegenerative and neurodevelopmental disorders described in association with the PURA protein. However, since most of the neuronal transport studies to date were performed in mice (Li et al., 2001; Ohashi et al., 2002; Kanai et al., 2004; Mitsumori et al., 2017), resulting findings need to be investigated further before any conclusions can be drawn about possible correlations with human disease-related processes.

## RNA-Repeat Expansion Disorders

PURA has been implicated in two different nucleotide-repeat expansion disorders: fragile X-associated tremor/ataxia syndrome (FXTAS) and the disease continuum of C9orf72-mediated amyotrophic lateral sclerosis and fronto-temporal dementia (C9 ALS/FTD).

### PURA in Hexanucleotide-Repeat Expansions in C9orf72 RNA Leading to ALS/FTD Spectrum Disorder

Current literature suggests that ALS and FTD are different phenotypic expressions of the same disease origin. Pathological expansions of up to thousands of G_4_C_2_ repeats in the first intron of the *C9orf72* gene have been described as the most frequent genetic cause of ALS/FTD (Dejesus-Hernandez et al., 2011; Renton et al., 2011). Hallmarks of the disease spectrum include RNA foci and progressive neurodegeneration. These RNA foci are mainly nuclear and contain G_4_C_2_ repeat sequences as well as a number of proteins including PURA (Donnelly et al., 2013; Mizielinska et al., 2013).

Three G_4_C_2_-repeat-dependent disease pathways have been proposed. The most obvious potential pathomechanism is the loss-of-function of the *C9orf72* gene product through the repeat expansions. However, since *C9orf72 knockout* mice failed to display an ALS/FTD-related motor neuron disease phenotype (Lagier-Tourenne et al., 2013; Koppers et al., 2015; Atanasio et al., 2016; McCauley et al., 2020), this option is rather unlikely. The second and third options are both related to the C9orf72 RNA and rather difficult to distinguish experimentally (discussed in Swinnen et al., 2020). One of these options is based on the observation that Repeat Associated Non-AUG translation (RAN translation) occurs on G_4_C_2_ repeat RNAs, resulting in potentially pathological protein fragments (Zu et al., 2011). Whether those RAN-derived proteins are solely or partially responsible for the ALS/FTD pathogenesis is still heavily discussed. While no functional role of PURA protein in RAN translation has been reported, PURA was found in pathological RNA foci of ALS/FTD patients (Xu et al., 2013b; Stepto et al., 2014). This indicates an involvement of PURA in the third possible disease pathway, i.e., RNA toxicity (Swinnen et al., 2018).

Since repeat expansion-containing RNAs are readily transcribed, the RNA itself could have neurotoxic properties. Different disease mechanisms are hypothesized for RNA toxicity to be responsible for the ALS/FTD symptoms. Firstly, the recruitment of proteins into RNA foci could result in sequestration of proteins such as PURA that are important for neuronal function (Stepto et al., 2014). Secondly, repeat expansions in the C9orf72 RNA could interfere with mRNA-transport granules and thus with neuronal function (Burguete et al., 2015).

For both scenarios, the identification of proteins binding to nucleotide expansions (G_4_C_2_) is essential. Indeed, multiple studies have analyzed these G_4_C_2_-interacting proteins and many RNA-binding proteins have been identified. The overlap of proteins that appear in all studies is limited though, probably due to technical differences (Swinnen et al., 2020). In a comparative analysis of several studies (Donnelly et al., 2013; Mori et al., 2013; Xu et al., 2013b; Cooper-Knock et al., 2014; Haeusler et al., 2014) with different cell lines, only five proteins are found in at least three out of five analyses. These proteins include HNRNPH3, HNRNPH1, ILF2, MBP, and SFPQ (Haeusler et al., 2016). Interestingly, PURA is detected with high significance in a study where pull-down experiments with extracts from mouse cerebellum were performed (Cooper-Knock et al., 2014). In a second study with mouse brain and spinal cord, PURA was again co-purified with G_4_C_2_ repeat expansions (Rossi et al., 2015). In contrast, PURA was not identified in pull-down experiments with G_4_C_2_ RNA from neuronal SH-SY5Y or HEK293T cells (Mori et al., 2013; Cooper-Knock et al., 2014; Haeusler et al., 2014). Together, these findings suggest that PURA is recruited to disease-related G_4_C_2_ sequences either exclusively in the context of functional neuronal tissues or only in mice but not in human cells.

In cell culture as well as model organisms, neurotoxicity induced by overexpression of G_4_C_2_-repeat RNAs can be overcome by a simultaneous ectopic overexpression of PURA (Xu et al., 2013b; Swinnen et al., 2018). On one hand, this underlines the importance of PURA for ALS/FTD pathogenesis. On the other hand, it also supports the hypothesis that repeat expansions sequester RNA-binding proteins, leading to a loss of physiological function of the affected proteins. Conversely, FMRP *knock down* mitigates the *C9orf72*-dependent ALS/FTD phenotype (Burguete et al., 2015), indicating opposing modes of action for FMRP and PURA.

In summary, PURA appears to play an important role as G_4_C_2_ repeat RNA interactor in a pathological context (Swinnen et al., 2020). It remains to be shown if the recruitment into foci depends on specific repeat binding events by PURA or is based on its rather general interaction with nucleic acids when these are present at pathologically high concentrations.

### The Role of PURA in FXTAS

Fragile X-associated tremor/ataxia syndrome is a late-onset condition that is caused by abnormally high numbers of 55–200 CGG-repeats in the 5'UTR of the *FMR1* gene (Hagerman and Hagerman, 2016; Salcedo-Arellano et al., 2020). Patients with more than 200 repeats develop a different disorder, termed Fragile X-syndrome, which is based on a transcriptional shut-down of the entire *FMR1* gene locus. The pathological repeat-expanded RNAs expressed in FXTAS patients recruit various proteins including PURA into intra-nuclear inclusions (Iwahashi et al., 2006; Jin et al., 2007; Sofola et al., 2007). Described hallmarks of FXTAS are a loss of Purkinje cells, RAN-translated FMR-PolyG proteins, and nuclear inclusions (Buijsen et al., 2014; Boivin et al., 2018). A well-documented disease mechanism for FXTAS is that the CGG repeat-expanded FMR1 mRNA sequesters important RNA-binding proteins. This leads to disturbed RNA processing very similar to what has been described for G_4_C_2_ repeat expansions in *C9orf72* as cause of ALS/FTD. Furthermore, CGG-repeat RNA-induced neurotoxicity can be overcome by simultaneous overexpression of PURA (Jin et al., 2007; Boivin et al., 2018).

As already stated for G_4_C_2_ repeats, RNA interactions of PURA with CGG repeats were shown by different studies but proof for a strict sequence-specificity is still lacking to date. In fact, one particular RNA oligonucleotide lacking CGG repeats even showed 10-fold better binding to PURA *in vitro* than CGG repeats of identical size (Weber et al., 2016).

### Potential Mechanisms of Neuroprotection by PURA

In disease models with CGG- or G_4_C_2_-repeat expansion-induced neurotoxicity, the overexpression of PURA rescues their pathological phenotypes. These observations indicate that PURA sequestration might be responsible for neurotoxicity. On the other hand, PURA overexpression is also described to ameliorate neurotoxicity of ALS related *FUS* mutations that are unrelated to RNA repeat expansions (Daigle et al., 2016). Here, PURA overexpression reverses cytoplasmic mislocalization of mutant FUS protein and enhances stress granule dynamics. The sequestration model is additionally challenged by the fact that multiple proteins are sequestered to repeat expansions but that the individual overexpression of PURA already reduces neurotoxicity (Jin et al., 2007; Xu et al., 2013a; Swinnen et al., 2018). One would expect that at least some of the other sequestered proteins also need to be released in order to restore normal neuronal function. Hence, the sum of these findings does not provide strong support for a disease-causing sequestration of PURA, rather hinting at other disease mechanisms such as an overall neuroprotective effect of PURA.

One mechanistic feature of PURA that could be important for such a neuroprotective function is its ability to unwind double-stranded nucleic acids in an ATP-independent manner (Darbinian et al., 2001a; Wortman et al., 2005). This function could help PURA to disassemble pathological RNPs and release trapped and mislocalized RNAs and proteins. A recent crystal structure of the N-terminal PUR domain bound to DNA provided a rationale for the molecular principles underlying such unwinding events (Weber et al., 2016). The structure shows that the two nucleic-acid binding β-sheets of a PUR domain form a wedge-like structure that seem suitable for intercalating between two breathing strands of double-stranded DNA (dsDNA) by contacting one strand and displacement of the corresponding complementary strand (Weber et al., 2016). It should be noted that also other members of the PC4-like protein family such as the Whirly protein WHY2 from plants are able to melt dsDNA (Cappadocia et al., 2010). Since NMR and *in vitro*-binding data indicated that PURA binds DNA in the same way as RNA (Weber et al., 2016), it is likely that PURA also unwinds dsRNA. This feature could help to disassemble pathological aggregates caused by tri-nucleotide expansion RNAs. Of note, in *Drosophila* PURA protein interacts with the RNA helicase RM62, which could further support the aggregate-dissolving function of PURA (Qurashi et al., 2011). Even more, the RM62 with a gain of function mutation is able to alleviate repeat-expansion RNA induced neurotoxicity even in absence of PURA. In summary, an appealing explanation for the neuroprotective function of PURA may lie in its unusual nucleic-acid binding and unwinding features.

## *Knockout* of *Pura* in Mice and its Implications

Consistent with an important and potentially neuroprotective function of PURA are the reported phenotypes of *Pura knock out* mouse models and, more recently, symptoms reported for a genetic human disorder, termed PURA syndrome.

**Figure 5:**
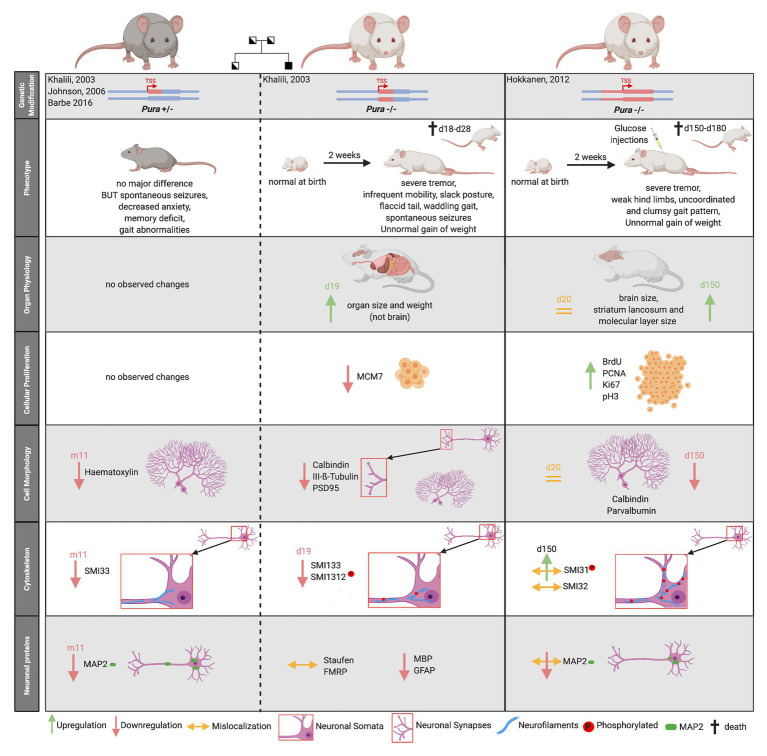
Comparison of two published Pura *knock-out* mouse models. Heterozygous mouse models are indicated by gray fur color of the depicted mice, homozygouse by white fur color. The deleted genomic region around the transcription start site (TSS) is highlighted by red color. The mouse models are not only characterized by their clinical phenotype but also by their organ physiology. Specific biomarkers were used to study cellular proliferation, cytoskeleton, and the overall cell morphology in mouse brain. Proliferation markers: MCM7, BrdU, PCNA, and Ki67. Neurofilament markers: SMI33, SMI32 = non-phosphorylated neurofilament marker, SMI1312, SMI31 = phosphorylated neurofilament marker, Purkinje cell marker = Calbindin, and Neuronal Marker: Class III-ß-Tubulin. Synapse Marker in CA3 region = PSD95, Neuronal dendritic marker: Parvalbumin, MAP2, Unspecific nuclear staining = Hematoxylin. “d” indicates days, “m” indicates months after birth.

The first evidence for a crucial role of PURA in postnatal neurodevelopment was provided in 2003 by Khalili et al. (2003) with the targeted disruption of the *Pura* gene in a mouse model. While a second *knockout* mouse model from an independent research group confirmed the main phenotype (Hokkanen et al., 2012), both studies differed significantly in several aspects (Figure 5).

Both homozygous *Pura knockout* mice seemed normal at birth but developed a similar neurological phenotype 2 weeks after birth. Mice suffered from a continuous and increasingly severe tremor upon motion, infrequent mobility, spontaneous seizures, and flaccid tail. They were unable to walk normally, showing abnormal gait pattern with clumsy and uncoordinated movements and weak hind limbs (Khalili et al., 2003; Hokkanen et al., 2012). *Pura*^−/−^ mice displayed feeding problems and did not gain normal weight (Khalili et al., 2003; Hokkanen et al., 2012). Without additional injections of glucose, they died within 4 weeks after birth (Hokkanen et al., 2012). Although the two research groups described a similar overall phenotype for their *knockout* mice, their histological analyses differed considerably.

Histological analysis of *knockout* mice from Khalili et al. (2003) revealed that multiple organs, but not the brain, were smaller and lighter than those of *wild type* mice. Their initial proliferation assays revealed decreased cellular proliferation in spleen, thymus, and hippocampal as well as cerebellar brain regions. Contrary to this report, the *knockout* mice by Hokkanen et al. (2012) showed megalencephaly with a significant enlargement of the stratum lancunosum and the molecular layer in the dentate gyrus of the brain. Cerebellum and hippocampus showed higher proliferation rates at some time points during development (Hokkanen et al., 2012). Neither study observed a compensation for differences in proliferation by adapted apoptosis (Khalili et al., 2003; Hokkanen et al., 2012). As summarized in Figure 5, both mouse models revealed alterations in the cytoskeleton of neurons, in Purkinje cell morphology as well as synapse formation, albeit with numerous inconsistent details at the histological level (Khalili et al., 2003; Hokkanen et al., 2012).

One common histological finding in both models was the significant reduction of the dendritic protein MAP2 during brain development and adolescence of mice (Figure 5). Hokkanen et al. (2012) additionally described a misdistribution of MAP2 in somata, leading to a loss of dendritic MAP2 protein.

The partially conflicting observations made in the different *Pura knock out* mouse models could have many reasons; including differences in the way the *knockout* mice were generated, different genetic backgrounds, and the use of different diagnostic tools. While Khalili et al. (2003) deleted 418 nucleotides of the *Pura* ORF directly 3' to the transcription start site (TSS) of embryonics stem (ES) cells derived from the mouse strain 129, Hokkanen et al. (2012) replaced the complete ORF of *Pura* and in addition 1.8 kb of the 3'-flanking sequence by homologous recombination in 129/Ola mouse ES cells (Figure 5, upper panel). Whereas in the first case, the knock-out strategy and validation is described rather briefly, Hokkanen et al. (2012) provide a more detailed description of follow up and validation experiments. One remarkable example for the influence of different diagnostic tools on their interpretation is their contradicting statements on cell proliferation. While Khalili et al. (2003) initially used a single marker to assess cell proliferation, Hokkanen et al. (2012) re-analyzed proliferation with four different markers, each specific for one cell cycle phase. It should also be considered that in the first published mouse model, the animals were unable to reach maturity and all observations were made in mice with a maximum age of 19 days (Khalili et al., 2003). In contrast, animals of the second *Pura*^−/−^ model received glucose injections during postnatal days 12–20 to be kept alive. As a consequence, they could be raised to adolescence (day 150) and respective observations be reported (Hokkanen et al., 2012).

Based on the mouse model of Khalili et al. (2003), heterozygous *Pura*^+/−^ mice were subsequently also characterized (Figure 5). This allowed to assess features that more directly relate to patients with heterozygous mutations in *PURA* (see below). In contrast to homozygous *Pura knockout* mice, almost no obvious morphological differences could be observed compared to their *wild type* littermates (Barbe et al., 2016). In heterozygous mice, only the number of neurons and dendrites seemed to be reduced and there was a higher prevalence of spontaneous early deaths (Barbe et al., 2016). Moreover, the heterozygous *Pura* animals showed decreased escape to touch, gait abnormalities, limb and abdominal hypotonia as well as significant memory deficits (Barbe et al., 2016). Such mouse models can potentially provide further insights into the pathogenesis of the equivalent human monogenetic neurodevelopmental disorder, PURA syndrome.

## The Neurodevelopmental Disorder Pura Syndrome

*PURA* first became a candidate for neurodevelopmental disorders when deletions of the genomic region 5q31.2–3, which includes the genes *Neuregulin2* (Ring et al., 1999) and *PURA* (Ma et al., 1995), were reported to correlate with intellectual disability (ID) and other related symptoms (Shimojima et al., 2011; Hosoki et al., 2012). Patients with this so-called 5q31.3 microdeletion syndrome displayed neurological abnormalities during postnatal development. In 2014, direct proof was published that heterozygous mutations in the *PURA* gene can lead to neurodevelopmental abnormalities (Hunt et al., 2014; Lalani et al., 2014). Patients with this disorder, named PURA syndrome, share several symptoms with patients developing the 5q31.3 microdeletion syndrome, suggesting that the lack of PURA accounts for many of the clinical features observed in the 5q31.3 microdeletion syndrome (Reijnders et al., 2017). While the 5q31.3 microdeletion syndrome was only reported in very few patients worldwide (Shimojima et al., 2011; Hosoki et al., 2012; Kleffmann et al., 2012; Brown et al., 2013), the number of patients diagnosed with PURA syndrome is increasing steadily (see below).

PURA syndrome is a sporadic monogenetic disorder and was first identified *via* whole exome sequencing. Since the initial description of 15 patients with PURA syndrome in 2014 (Hunt et al., 2014; Lalani et al., 2014), a few hundred have been diagnosed around the globe (personal communication: PURA Syndrome Foundation). It is therefore classified as a rare disorder. Patients are usually diagnosed by gene-panel testing or whole exome sequencing (Reijnders et al., 2018; Jezela-Stanek et al., 2020). Of note, the latter shows poorer coverage at the 5'end of the *PURA* open reading frame (https://gnomad.broadinstitute.org/; Karczewski et al., 2020), which is possibly due to the low sequence complexity and high GC content of this region that might interfere with the reverse transcription step of this method. This observation suggests an increased risk of missing disease-causing mutations when whole exome sequencing is used exclusively for diagnosis. *PURA* is now included in many developmental disorder and epileptic encephalopathy gene panels, meaning that the diagnosis of PURA syndrome can be made much more readily than when the disorder was first described in 2014.

**Figure 6:**
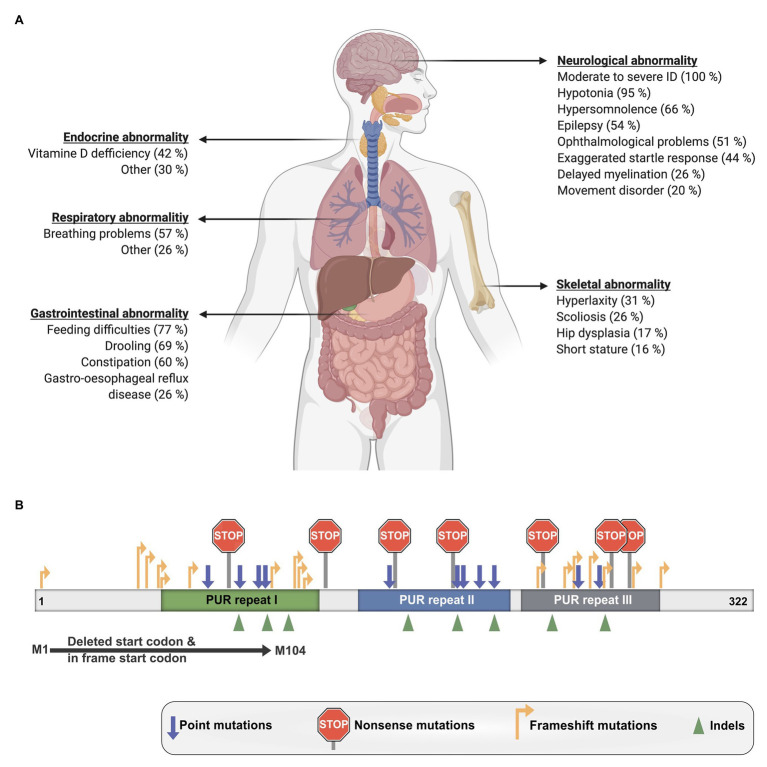
Clinical manifestations of PURA syndrome and pathogenic mutations. **(A)** Clinical manifestations in PURA-syndrome patients and their relative frequencies. **(B)** Mutations causing PURA syndrome are scattered over most parts of the *PURA* open reading frame. Information presented in this figure is based on previously published data (Reijnders et al., 2018).

The disorder is characterized by a mild to moderate developmental delay and ID as well as numerous other clinical manifestations that vary considerably between patients (Figure 6A; Reijnders et al., 2018). Nearly all investigated affected individuals suffered from hypotonia, feeding difficulties, and remained non-verbal. Around half of the patients also exhibited gastrointestinal problems such as constipation and drooling as well as breathing problems, hypersomnolence, ophthalmological abnormality, and epilepsy. Additional reported symptoms were skeletal and respiratory issues as well as endocrine abnormalities like Vitamin-D deficiency. Furthermore, one case of hypoglycorrhachia (Mayorga et al., 2018), cutis laxa (Cinquina et al., 2020), and symptoms resembling infantile hypotonia with psychomotor retardation and characteristic facies (IHPRF; Mishra et al., 2021) have been recently reported.

Given the recent discovery of the PURA syndrome in 2014, only a limited number of systematic clinical studies have been reported to date. MRI brain scans of affected children showed a frequent dysmaturation of the cortical white matter due to delayed and decreased myelination (Hunt et al., 2014; Tanaka et al., 2015; Lee et al., 2018). A recent case study of an individual patient also showed an enlargement of the brain stem (Rodriguez-Garcia et al., 2020). It is obvious that more clinical studies are required to address the functional implications of such morphological differences. Along these lines, a clinical, EEG-based study has been recently established with support of the PURA Syndrome Foundation.^1^

While these first studies on the CNS point toward certain morphological abnormalities, to our knowledge, no significant work has been undertaken to study the peripheral nervous system. For instance, the hypotonic symptoms of patients could be caused either by insufficient signaling from the central nervous system or by defects in the peripheral nervous system, including neuro-muscular junctions or even in target structures of the peripheral nervous system such as muscle cells. Indeed, two recent case studies support a muscular contribution to the symptoms. Whereas the first study reported a PURA-syndrome patient with electrical myotonia with hypotonia as clinical symptom (Trau and Pizoli, 2020), the second study provides direct evidence for myopathic changes, including fiber size variability and fast fiber atrophy as main features (Mroczek et al., 2020).

There is also molecular evidence for a PURA-dependent impairment of muscle cells, as PURA and PURB were recently shown to contribute to differentiation of myotubes (Gupta et al., 2003; Ji et al., 2007; Hariharan et al., 2014; Pandey et al., 2020). For a clearer picture, electro-physiological work will have to go hand in hand with other approaches, including postmortem histological analyses of neuronal and muscular tissues as well as functional studies, to understand the respective contribution of different tissues to the observed symptoms in patients.

To date, also the molecular pathomechanisms leading to PURA syndrome have not been understood sufficiently. Heterozygous dominant mutations like those in *PURA* are usually thought to result in functional haploinsufficiency. Already in the original description by the United Kingdom-based initiative Deciphering Developmental Disorders (DDD) study haploinsufficiency was strongly suggested for PURA syndrome (Huang et al., 2010; Deciphering Developmental Disorders Study, 2015). A more direct support for haploinsufficiency as disease-causing mechanism is the observation that no correlation is found between the type of mutation and the specific symptoms of patients. A mutation disrupting the start codon of the intron-less *PURA* gene as well as frameshift mutations at the very 5' end of the open reading frame or various frameshift, missense, and nonsense mutations located more 3' of the gene all lead to the full spectrum of symptoms (Figure 6B; Reijnders et al., 2018). Most likely all of these mutant PURA proteins are non-functional and the remaining wild-type allele of *PURA* produces insufficient amounts of protein to fulfill its cellular function. Considering that PURA protein forms homodimers (Figures 1A,B; Graebsch et al., 2009; Weber et al., 2016), a mutant protein that still dimerizes could potentially even form inactive complexes with the healthy copy of PURA, thereby leading to a 75% loss of functional PURA dimers.

Some disorders such as the Rett syndrome show symptoms that resemble aspects of the PURA syndrome. This resulted in misdiagnosis of some PURA-syndrome patients prior to the availability of genetic testing. Rett syndrome is caused by mutations in the *MECP2* gene (Tillotson and Bird, 2019; Sandweiss et al., 2020), where disease-causing hot-spot regions have been described (Lombardi et al., 2015). Furthermore, truncation mutations located in more 5' regions of the *MECP2* gene show more severe phenotypes in patients with Rett syndrome than in patients with more 3'-located mutations (Lombardi et al., 2015). Also, for the PURA syndrome, an analysis of mutations was performed, in which the location of mutations within the gene was compared with the severity of symptoms. In contrast to mutations in *MECP2*, PURA syndrome-causing mutations are spread almost over the entire *PURA* sequence (Figure 6B; Reijnders et al., 2018). Only mutations in the unstructured N- and C-terminal regions and a few conservative amino-acid exchanges within the folded domains do not result in PURA syndrome. In contrast, almost all mutations in folded domains results in the full-blown, variable spectrum of clinical features observed in PURA patients. Only few recurrent mutations were reported, the most extreme example is F233del with over a dozen of cases (personal communication: PURA Syndrome Foundation). Surprisingly, even for those patients with identical mutations, the full variation in penetrance of individual phenotypes could be observed (Reijnders et al., 2018). This observation points at potential modulators of the function of PURA, such as its interacting proteins or different levels of regulation of downstream targets. In summary, there is no obvious connection between the type and location of a given mutation within the open reading frame and its specific clinical phenotype.

The observed severe defects by a large range of mutations along most parts of the protein together with structural considerations suggest that PURA protein is very sensitive to changes. This is indeed reflected in the high sequence conservation of this protein (Figure 1D).

## Discussion

PURA syndrome is caused by heterozygous *de novo* mutations within the *PURA* gene. These mutations have been suggested to result in a functional haploinsufficiency leading to a variable spectrum of phenotypes (Figure 6A; Hunt et al., 2014). When comparing the phenotype of the *Pura knockout* mice with PURA syndrome patients, a number of similarities can be found. For instance, homozygous *knockout* mice did not show any phenotype until 2 weeks postnatal. Subsequently, they developed a continuous and increasingly severe tremor, weak hind limps, and uncoordinated gait pattern (Khalili et al., 2003; Hokkanen et al., 2012). Also in patients, similar symptoms were reported (Figure 6A). In mice, epileptic seizures have only been described by Khalili et al. (2003), but not for the other mouse model (Hokkanen et al., 2012). Despite these differences, PURA appears to be highly relevant for neurodevelopment as it can not only be seen in *knock out* mouse models but also in patients that contain mutations in one copy of the *PURA* gene. The association with neuronal mRNA transport and translation connects PURA to several other disorders that are described to originate mainly from dysfunctions of these processes. This includes neurodevelopmental as well as neurodegenerative disorders such as ALS/FTD and FXTAS. Also, other proteins described to be involved in these RNA related processes have been linked to human disorders. Examples are different Kinesin motors, Ataxin-2, MBNL, and TDP-43, among others (Wang et al., 2016; Engel et al., 2020; Kalantari and Filges, 2020; Zhao et al., 2020). Nevertheless, it should be taken into account that *PURA* is ubiquitously expressed in the human body and might fulfill important functions in a range of different tissues (Human Protein Atlas, available from http://www.proteinatlas.org; Uhlen et al., 2015).

Hence, it will not only be necessary to understand which role PURA plays in neural cells. We also need to thrive for a better understanding of the role of PURA in non-neural tissues as this will help to elucidate systemic disease processes in patients with PURA syndrome.

## Author Contributions

LM, SBa, SBu, and DN designed, discussed and wrote the manuscript. All authors contributed to the article and approved the submitted version.

### Conflict of Interest

The authors declare that the research was conducted in the absence of any commercial or financial relationships that could be construed as a potential conflict of interest.

## Footnotes

^1^https://www.purasyndrome.org/eeg
